# Optimization of large animal MI models; a systematic analysis of control groups from preclinical studies

**DOI:** 10.1038/s41598-017-14294-z

**Published:** 2017-10-27

**Authors:** P. P. Zwetsloot, L. H. J. A. Kouwenberg, E. S. Sena, J. E. Eding, H. M. den Ruijter, J. P. G. Sluijter, G. Pasterkamp, P. A. Doevendans, I. E. Hoefer, S. A. J. Chamuleau, G. P. J. van Hout, S. J. Jansen of Lorkeers

**Affiliations:** 1 Department of Cardiology, University Medical Center Utrecht, Utrecht, The Netherlands; 2 Center for Clinical Brain Sciences, University of Edinburgh, Edinburgh, United Kingdom; 3 Hubrecht Institute, Koninklijke Nederlandse Academie van Wetenschappen (KNAW), University Medical Center Utrecht, Utrecht, The Netherlands; 4 Netherlands Heart Institute (ICIN), Utrecht, The Netherlands; 5 UMC Utrecht Regenerative Medicine Center, University Medical Center Utrecht, Utrecht, The Netherlands; 6 Central Military Hospital, Utrecht, The Netherlands; 7 Department of Clinical Chemistry and Hematology, University Medical Center Utrecht, Utrecht, The Netherlands

## Abstract

Large animal models are essential for the development of novel therapeutics for myocardial infarction. To optimize translation, we need to assess the effect of experimental design on disease outcome and model experimental design to resemble the clinical course of MI. The aim of this study is therefore to systematically investigate how experimental decisions affect outcome measurements in large animal MI models. We used control animal-data from two independent meta-analyses of large animal MI models. All variables of interest were pre-defined. We performed univariable and multivariable meta-regression to analyze whether these variables influenced infarct size and ejection fraction. Our analyses incorporated 246 relevant studies. Multivariable meta-regression revealed that infarct size and cardiac function were influenced independently by choice of species, sex, co-medication, occlusion type, occluded vessel, quantification method, ischemia duration and follow-up duration. We provide strong systematic evidence that commonly used endpoints significantly depend on study design and biological variation. This makes direct comparison of different study-results difficult and calls for standardized models. Researchers should take this into account when designing large animal studies to most closely mimic the clinical course of MI and enable translational success.

## Introduction

Large animal studies are needed to test therapeutic efficacy of novel therapies for myocardial infarction (MI). These studies usually serve as crucial checkpoints before advancing to first-in-man trials^1,2^. Considerable heterogeneity exists in the models currently used to study MI and its aftermath^3^. The choice for a specific model may influence the manifestation and progression of the disease and subsequently the potential effect of an intervention or technique under evaluation^3^.

There is a strong demand for optimal selection of models that represent the human disease best, since many promising therapeutics have shown beneficial effects in the preclinical phases, but fail in the clinical setting^4^. Methodological flaws and inadequate modeling of human MI have been proposed as partial explanations of this ‘translational failure’, leading to false positive study outcomes and the risk of overestimation of effect size in preclinical studies^5–8^. However, systematic analysis of methodological decisions on effect size are currently not available.

Standardization of these animal models could be of value for comparison of individual studies to historical data, for which groups in the field of cardioprotection have put forth the first efforts^2,9^. Above all, the translational value of large animal MI models can be significantly increased by assessing the effect of model design on primary outcome. This enables selection of animal models that most resemble the clinical course of MI.

**Figure 1:**
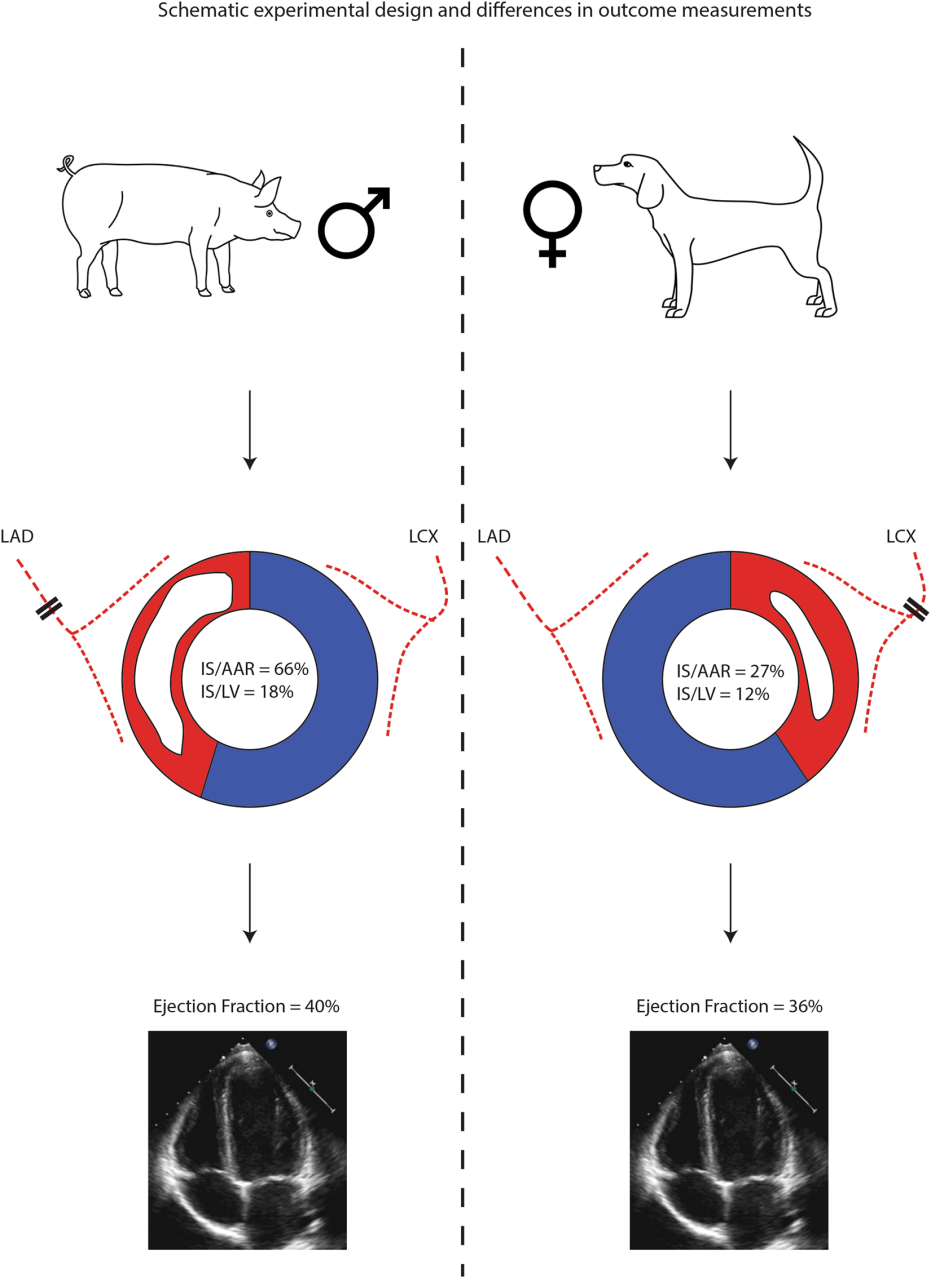
Graphical schematic representation of differences in outcomes after MI through study design - A model using male pigs in combination with an LAD-occlusion will differ significantly from a female dog model with LCX-occlusion.

In the evolving era of big data and abundant publication, the research community is calling on meta-research to systematically evaluate and improve research methods^10,11^. Systematic reviews and meta-analyses of preclinical data not only provide us with comprehensive overviews and bias assessments, but can also provide us with additional insights that explain heterogeneity within a specific disease and intervention^12^. In this perspective, combining and examining control groups of preclinical studies for a certain disease model, provides us with a comprehensive data-heavy method of studying the progression of the disease model and quantify the potential influence of certain variables on standard disease outcomes. The aim of the current study was to systematically explore the natural course of artificially induced MI in different large animal models and ultimately determine which biological and methodological factors act as effect modifiers, influencing disease course, primary endpoints and mortality within studies. Through meta-analysis, we report that functional and anatomical endpoints following MI in large animal models vary significantly due to variability in study design (Fig. 1).

## Methods

Data from control animals from two previous meta-analyses on large animal MI models were collected^7,8^. In both datasets infarct size as a ratio of the area at risk (IS/AAR), infarct size as a ratio of the left ventricle (IS/LV) and left ventricular ejection fraction (EF) were extracted and added in the current data if not present. Results on peri- and post-procedural mortality were extracted for all studies; peri-procedural meaning within the timeframe of the infarct-induction process (‘death during surgical procedures’) and post-procedural meaning after the disease-inducing procedure. Any procedural complications not due to the induction of the MI itself were not counted as ‘natural’ mortality. Due to evolving methodology over time in MI modeling with regards to the treatment of ventricular fibrillation (VF) during induction of MI, we recorded whether animals were treated for VF (either by medication or defibrillation) or were excluded immediately and performed a predefined sensitivity analysis to exclude a potential effect of this specific early exclusion. A thorough explanation of methodology on mortality data extraction can be found in the Supplementary section.

Pre-defined variables of interest were species, sex, age, weight, use of immunosuppression, co-medication commonly used in clinical care of MI (defined as being treated for the whole study after MI with one or more of the following compounds: aspirin, clopidogrel, ticagrelor, prasugrel, beta-blockers, ACE-inhibitors, angiotensin receptor blockers, and/or statins), follow-up duration post-MI, study quality and multiple characteristics of the infarct induction procedure: open-thorax vs closed percutaneous procedure, permanent vs temporary occlusion, ischemia duration (if transient occlusion) and type of vessel occluded (left coronary artery (LCA) vs left circumflex artery (LCX) vs left anterior descending (LAD) vs right coronary artery (RCA)). The variable method of quantification (for infarct size measurement or ejection fraction) was added in the phase of revisions to correct for any effect of these methods on regular outcomes. Study quality was assessed using the ‘Collaborative Approach to Meta Analysis and Review of Animal Data from Experimental Studies’ (CAMARADES) quality checklist^13^. As data on age and weight was scarcely available in the included studies, we conducted a post-hoc sensitivity analysis between minipigs and regular pigs within our species variable, as these substantially differ with regards to total body weight and age. All studies that did not report the strain of pigs were pooled in an ‘unreported’ variable.

Any variable not already assessed prior to this project, was added to the database.

All data has been inserted in the CAMARADES database (available on request)^14^.

### Statistical analysis

Random effects meta-analysis with restricted maximum likelihood was performed due to anticipated heterogeneity between the different models of disease. Forest plots were generated to visualize these. Correlation analysis was performed between IS/AAR and EF using linear regression. Correlation between the actual therapeutic effect of included studies and the values of control animals was also assessed using linear regression.

Univariable meta-regression was performed for the association of chosen variables with our outcomes of interest. All variables were subsequently tested in multivariable meta-regression with the outcomes IS/AAR, IS/LV, EF and mortality, to correct for potential effect modification and to distinguish independent effects. Of note, multivariable meta-regression is especially suitable in the setting of animal studies, as all variables of interest are deliberately kept constant in preclinical study setup as opposed to the clinical setting. This minimalizes the risk of a potential ecological bias in our analysis. A post-hoc Wald test was used for categorical univariable meta-regression with more than two categories and in multivariable meta-regression to determine the individual association per individual variable. We used raw means for the outcomes IS/AAR, IS/LV and EF, since percentages are not expected to differ between the different groups under study.

For mortality outcomes, we used ratios (number of dead animals per total animals) and weighed each measurement on the inversed square root of the total number of animals for each comparison in our meta-regression analysis (1/√n). In the case of two measurements in the same procedural setting (for example mentioning of mortality peri-procedural both before and after randomization), the appropriate ratio was determined by multiplying both proportions (1 − p_total_ = (1 − p_1_) * (1 − p_2_)). The weighing factor for such a value is the square root of the total number of animals in both measurements, divided by two (1/√((n_1_ + n_2_)/2)). A p-value of <0.05 was considered significant.

For our prediction modeling strategy, we used multivariable meta-regression to predict the outcomes for commonly used large animal models. We modeled both a pig and a dog model of temporary 60-minute occlusion with follow-up of 1 day, 1 week and 1 month. We did the same for a chronic occlusion pig model, using the same follow-up times. Statistical analyses were performed using R version 3.1.2^15^ with the additional metafor package^16^ and Stata version 11 (Statacorp, LP, Texas, USA). The R script is available in the Supplementary section.

## Results

A total of 246 studies were used, yielding 1500, 1221 and 775 animals for the outcomes IS/AAR, IS/LV and EF, respectively (Table 1). For the mortality analyses, data of 3622 animals and 1555 animals was studied for peri-procedural and post-procedural mortality, respectively (Table 1).

**Table 1:** Number of included animals per dataset.

	Datasets			
	Jansen of Lorkeers *et al*.^7^	van Hout *et al*.^8^	This meta-analysis	Average outcome (MA)
*IS/AAR*	0	1500	1500	49.8%
*IS/LV*	261	960	1221	18.1%
*Ejection Fraction*	584	191	775	39.3%
*Peri-procedural mortality*	1183	2439	3622	16.7%
*Post-procedural mortality*	365	1190	1555	5.2%

MA = meta-analysis.

### Meta-analysis

From our datasets, an average IS/AAR of 49.8% (95%CI 46.0–53.6%), IS/LV of 18.1% (95%CI 16.5–19.7%) and EF of 39.3% (95%CI 37.4–41.2%) were observed after MI induction and follow-up (Table 1). These outcomes are also visualized in Forest Plots (Supplementary Figures 1–3). The average peri-procedural mortality and post-procedural mortality were 16.7% (95% CI 14.7–18.7%) and 5.2% (95% CI 3.6–6.9%) respectively (Table 1).

### Correlation between assessed outcomes

To study the effect of the initial damage and therapeutic effect of any drug given, we used linear regression to compare the absolute therapeutic effect within a study and the mean outcome that was assessed in the control animals. For IS/AAR (p = 0.0001), IS/LV (p = 0.001) and EF (p = 0.05) there was a significant correlation between the effect of the study therapeutic and the initial damage in the control animal (Supplementary Figure 4A–C). This indicates that greater cardiac damage leads to a larger effect of the investigated therapeutic.

There was no correlation observed between IS/AAR and EF if measured in the same study (p = 0.66, Supplementary Figure 5).

### Meta-regression on standard outcomes: IS/AAR

Univariable meta-regression revealed multiple correlating variables with all our outcomes (Tables 2–4), which were subsequently used for multivariable analyses.

Multivariable meta-regression (p < 0.001) for the outcome IS/AAR revealed that infarct size was smaller when **dogs** were used (−22% compared to **pigs** (p < 0.001)). **Male animals** were also at risk for larger infarcts (−6% for both sexes compared to male (p = 0.040) and −10% for unreported sexes compared to male (p = 0.010)). The use of **co-medication** was protective (−18% if used (p = 0.01)) and infarct size was also dependent on the **type of occlusion** (−36% if temporary compared to permanent occlusion (p < 0.001) and −46% if temporary compared to unknown occlusion (p < 0.001)). Occlusion of the LAD leads to larger IS/AAR (+8% compared to LCX (p = 0.008)) and **follow-up duration** (−0.3% per hour of follow-up (p = 0.011)) also independently influenced the outcome (Table 2). For all temporary occlusion studies (n = 145), **ischemia duration** was an additional significant influencing variable in multivariable meta-regression of IS/AAR (+0.09%/min ischemia (p = 0.001)) (Table 2).

**Table 2:** Univariable and multivariable meta-regression for outcome IS/AAR.

Univariable Analysis						Multivariable analysis			
Variable	categories	n	mean (95%CI)	p-value	post-hoc p-value	Variable	p-value	beta	post-hoc p-value
Species	Dog	122	46.4 (43.1–49.7)	<**0.001**	**0.001 (pig vs dog)**	Species	<**0.001**	+**21.6 if pig (vs dog)**	<**0.001**
	Pig	41	59.3 (53.6–65.0)		0.552 (pig vs sheep)			+1.8 if pig (vs sheep)	0.874
	Sheep	2	67.5 (41.0–93.9)		0.120 (dog vs sheep)			−19.8 if dog (vs sheep)	0.071
Sex	Male	45	47.7 (41.9–53.4)	0.80	All comparisons NS	Sex	0.07	+10.5 if male (vs female)	0.114
	Female	11	50.0 (38.4–61.6)					+**10.4 if male (vs unknown)**	**0.010**
	Both	78	51.3 (46.9–55.6)					−0.1 if female (vs unknown)	0.990
	Unknown	31	49.5 (42.6–56.4)					+**6.3 if male (vs both)**	**0.040**
								−4.1 if female (vs both)	0.491
								+4.0 if both (vs unknown)	0.255
Immunosupp		*not applicable*				Immunosupp	*not applicable*		
Comedication	yes	7	43.6 (29.2–58.0)	0.379		Comedication	**0.01**	−18.2 if used	
	no	158	50.2 (47.1–53.2)						
Open vs closed model	Open	129	50.3 (46.9–53.7)	0.536	0.293 (open vs closed)	Open vs closed model	0.22	+4.9 if open (vs closed)	0.130
	Closed	35	46.7 (40.2–53.3)		0.745 (open vs unknown)			−13.1 if open (vs unknown)	0.433
	Unknown	1	57.0 (18.7–95.3)		0.603 (closed vs unknown)			−17.9 if closed (vs unknown)	0.287
Occlusion	Permanent	17	69.1 (60.5–77.7)	<**0.001**	<**0.001 (permanent vs temporary)**	Occlusion	<**0.001**	+**36.3 if permanent (vs temporary)**	<**0.001**
	Temporary	145	47.2 (44.2–50.2)		0.072 (permanent vs unknown)			−9.2 if permament (vs unknown)	0.343
	not known	3	65.9 (45.8–86.0)		0.774 (temporary vs unknown)			**−45.7 if temporary (vs unknown)**	<**0.001**
Occluded vessel	LAD	108	54.3 (50.8–57.7)	<**0.001**	<**0.001 (LAD vs LCX)**	Occluded vessel	**0.03**	+**7.6 if LAD (vs LCX)**	**0.008**
	LCX	53	40.6 (35.6–45.6)		0.921 (LAD vs LAD/LCX)			+2.2 if LAD (vs LAD/LCX)	0.788
	LAD/LCX	4	55.2 (36.9–73.5)		0.13 (LCX vs LAD/LCX)			−5.4 if LCX (vs LAD/LCX)	0.523
Quantification method	TTC	140	49.1 (45.8–52.3)	0.30	All comparisons NS	Quantification method	0.17	All comparisons NS	
	Nitro blue	10	60.6 (48.7–72.5)						
	Planimetry	11	48.6 (37.2–60.0)						
	Other^§^	4	54.1 (35.2–72.9)						
Follow-up duration		165	−0.02/hour (−0.05–0.01)	0.12		Follow-up duration	**0.011**	**−0.03/hour**	
Study Quality		165	+0.36/point (−1.7–2.4)	0.734		Study quality	0.945	+0.07/point	
Ischemia time		145	−0.01/min (−0.07–0.05)	0.723		Ischemia time*(*n* = *56*)	**0.001**	+**0.09/min**	
Weight		159	+0.48/kg (0.219–0.743)	<**0.001**		Weight*(*n* = *1*5*9*)	0.124	+0.25/kg	
Age		*5*	−0.25/wk (−3.24–2.74)	0.806		Age*(*n* = *5*)	*not applicable*		

^§^MRI, Fluoroluminescence, NADH Fluorescence or 111ln-Antimyosin. NS = non-significant. *Variable was added to the multivariable model separately, due to missing data. Total multivariable meta-regression was significant (p < 0.0001). n = the number of comparisons (=165 in total).

### Meta-regression on standard outcomes: IS/LV

Multivariable meta-regression analysis (p < 0.001) for IS/LV showed that **occluded vessel** (p = 0.030) and method of quantification (p = 0.01) are of significant influence. For quantification methodology, MRI and planimetry underestimated infarct size compared to tissue staining and other modalities. Furthermore, **study quality** was associated with a 1.3% difference in IS/LV per quality point (Table 3). The variables **species** and **sex** showed only a trend (p = 0.05 and p = 0.08 respectively) for an association, with the same directions for categories as in the IS/AAR analyses (Table 3).

**Table 3:** Univariable and multivariable meta-regression for outcome IS/LV.

Variable	Univariable Analysis					Multivariable analysis			
	categories	n	mean (95%CI)	p-value	post-hoc p-value	Variable	p-value	beta	post-hoc p-value
Species	Dog	90	16.7 (14.9–18.5)	**0.040**	**0.015 (pig vs dog)**	Species	0.05	+**6.3 if pig (vs dog)**	**0.023**
	Pig	52	20.4 (18.0–22.8)		0.640 (pig vs sheep)			−5.4 if pig (vs sheep)	0.502
	Sheep	1	24.4 (7.7–41.1)		0.365 (dog vs sheep)			−11.7 if dog (vs sheep)	0.160
Sex	Male	35	19.6 (16.7–22.5)	0.247	All comparisons NS	Sex	0.08	+**6.5 if male (vs female)**	**0.047**
	Female	18	18.7 (14.6–22.8)					+**5.4 if male (vs unknown)**	**0.022**
	Both	50	16.0 (13.6–18.5)					−1.2 if female (vs unknown)	0.650
	Unknown	40	18.9 (16.2–21.6)					+**4.0 if male (vs both)**	**0.033**
								−2.6 if female (vs both)	0.402
								+1.4 if both (vs unknown)	0.492
Immunosupp	yes	3	12.1 (2.1–22.1)	0.236		Immunosupp	0.23	−5.9 if used	
	no	140	18.2 (16.7–19.7)						
Co-medication	yes	9	15.4 (9.4–21.3)	0.361		Co-medication	0.22	−4.0 if used	
	no	134	18.2 (16.7–19.7)						
Open vs closed model	Open	99	18.7 (16.9–20.4)	0.224		Open vs closed model	0.46	−1.5 if open model	
	Closed	44	16.7 (14.1–19.4)						
Occlusion	Permanent	46	20.2 (17.6–22.7)	0.138	0.047 (permanent vs temporary)	Occlusion	0.065	+**4.1 if permanent (vs temporary)**	**0.012**
	Temporary	95	17.0 (15.3–18.8)		0.677 (permanent vs unknown)			+0.5 if permament (vs unknown)	0.932
	not known	2	17.6 (5.4–29.7)		0.933 (temporary vs unknown)			−3.8 if temporary (vs unknown)	0.529
Occluded vessel	LAD	93	19.2 (17.4–20.9)	**0.004**	0.130 (LAD vs LCX)	Occluded vessel	**0.009**	+0.3 if LAD (vs LCX)	0.869
	LCX	47	16.8 (14.4–19.3)		**0.002 (LAD vs LAD/LCX)**			+**15.5 if LAD (vs LAD/LCX)**	**0.002**
	LAD/LCX	3	3.7 (−5.7–13.1)		**0.008 (LCX vs LAD/LCX)**			+**15.2 if LCX (vs LAD/LCX)**	**0.004**
Quantification Method	TTC	86	17.8 (16.0–19.7)	0.06	**0.007 (MRI vs other)**	Quantification Method	**0.012**	+**8.3 if Nitro Blue (vs Planimetry)**	**0.022**
	Nitro Blue	8	20.8 (15.1–26.5)		**0.008 (Planimetry vs other)**			+**10.1 if Nitro Blue (vs MRI)**	**0.019**
	Planimetry	22	16.7 (13.1–20.4)		**0.01 (TTC vs other)**			**−9.0 if Planimetry (vs Other)**	**0.026**
	MRI	19	16.5 (12.6–20.3)		Rest of comparisons NS			**−10.9 if MRI (vs Other)**	**0.005**
	Other^§^	7	27.5 (20.4–34.5)					Rest of comparisons NS	
Follow-up duration		143	+0.001/hour (−0.001–0.002)	0.565		Follow-up duration	0.326	−0.001/hour	
Study Quality		143	+1.52/point (0.67–2.37)	**0.001**		Study quality	**0.026**	+**1.3**/**point**	
Ischemia time		95	+0.002/min (−0.002–0.006)	0.414		Ischemia time*(*n* = *95*)	0.131	+0.003/min	
Weight		137	−0.006/kg (−0.17–0.16)	0.946		Weight*(*n* = *137*)	0.297	−0.10/kg	
Age		11	+0.05/wk (−0.14–0.25)	0.568		Age*(*n* = *11*)	*not applicable*		

^§^(SPECT−) CT, NOGA mapping or Masson’s Trichrome staining, NS = non-significant. *Variable was added to the multivariable model separately, due to missing data Total multivariable meta-regression was significant (p < 0.0001). n = the number of comparisons (=143 in total)

### Meta-regression on standard outcomes: EF

Multivariable meta-regression for EF showed an effect of **species**, with a 9% difference in EF for pigs compared to sheep (p = 0.007). **Sex** also independently influenced EF after MI (−6% for female animals compared to male animals (p = 0.03), −7% for female animals compared to studies using both sexes (p = 0.028) and −6% for female animals compared to animals with unreported sex (p = 0.009)) (Table 4). The choice of **occluded vessel** also showed an independent effect (+24.2 for only an LAD occlusion (p = 0.014), +26.2 for only an LCX occlusion (p = 0.009) compared to a combined LAD/LCX occlusion); again, this should be interpreted with caution, as the number of comparisons using either the LAD or LCX in the same study is limited (Table 4). **Method of quantification** had an independent effect on ejection fraction outcome, with echocardiography estimating higher ejection fraction values compared to LV Angio (+6.7%, p = 0.030), SPECT (+7.7%, p = 0.034) and PV loop (+12.6%, p = 0.006).

**Table 4:** Univariable and multivariable meta-regression for outcome ejection fraction.

Variable	Univariable Analysis					Multivariable analysis			
	categories	n	mean (95%CI)	p-value	post-hoc p-value	Variable	p-value	beta	post-hoc p-value
Species	Dog	15	36.5 (31.3–41.8)	**0.011**	0.144 (pig vs dog)	Species	**0.01**	+4.7 if pig (vs dog)	0.151
	Pig	87	40.7 (38.6–42.8)		**0.005 (pig vs sheep)**			+**8.6 if pig (vs sheep)**	**0.007**
	Sheep	11	31.9 (26.1–37.7)		0.238 (dog vs sheep)			+3.9 if dog (vs sheep)	0.357
Sex	Male	21	37.9 (33.6–42.2)	0.068	0.398 (male vs female)	Sex	**0.04**	+**6.3 if male (vs female)**	**0.033**
	Female	27	35.4 (31.6–39.3)		0.206 (male vs unknown)			+0.2 if male (vs unknown)	0.943
	Both	15	39.4 (36.9–47.5)		0.018 (female vs unknown)			**−6.1 if female (vs unknown)**	**0.009**
	Unknown	50	41.2 (38.4–44.0)		0.214 (male vs both)			−0.9 if male (vs both)	0.789
					0.043 (female vs both)			**−7.2 if female (vs both)**	**0.028**
					0.741 (both vs unknown)			+1.1 if both (vs unknown)	0.702
Immunesupp	yes	6	37.4 (29.1–45.7)	0.658		Immunosupp	0.75	−1.3 if used	
	no	107	39.3 (37.1–41.5)						
Co-medication	yes	11	43.7 (37.6–49.9)	0.135		Co-medication	0.21	+4.0 if used	
	no	102	38.8 (36.8–40.8)						
Open vs closed model	Open	50	39.1 (36.2–42.0)	0.868		Open vs closed model	0.90	−0.3 if open model	
	Closed	63	39.4 (36.8–42.0)						
Occlusion	Permanent	56	36.5 (33.9–39.1)	**0.013**	**0.005 (permanent vs temporary)**	Occlusion	0.12	−4.2 if permanent (vs temporary)	0.064
	Temporary	55	41.9 (39.2–44.5)		0.175 (permanent vs unknown)			−8.2 if permanent (vs unknown)	0.257
	not known	2	46.5 (32.2–60.7)		0.531 (temporary vs unknown)			−4.0 if temporary (vs unknown)	0.581
Occluded vessel	LAD	89	41.2 (36.7–45.7)	**0.011**	0.618 (LAD vs LCX)	Occluded vessel	**0.03**	−2.0 if LAD (vs LCX)	0.389
	LCX	23	41.0 (32.4–49.6)		**0.003 (LAD vs LAD/LCX)**			+**24.2 if LAD (vs LAD/LCX)**	**0.014**
	LAD/LCX	1	10 (−8.1–28.1)		**0.003 (LCX vs LAD/LCX)**			**26.2 if LCX (vs LAD/LCX)**	**0.009**
Quantification Method	Echo	56	41.0 (38.4–43.6)	**0.04**	**0.014 (Echo vs PV loop)**	Quantification Method	**0.01**	+**6.7 if echo (vs LV Angio)**	**0.030**
	MRI	27	40.6 (36.8–44.4)		**0.022 (MRI vs PV loop)**			+**7.7 if echo (vs SPECT)**	**0.034**
	LV Angio	27	36.8 (32.0–41.6)		Rest of the comparisons NS			+**12.6 if echo (vs PV loop)**	**0.006**
	SPECT	8	40.0 (26.77–41.2)					Rest of the comparisons NS	
	PV loop	5	29.4 (20.6–38.2)						
Follow-up duration		113	−0.0002/hour (0–0.0003)	0.338		Follow-up duration	0.11	−0.0004/hour	
Study Quality		113	0.14/point (−1.4–1.7)	0.859		Study quality	0.19	−1.0/point	
Ischemia time		55	−0.04/min (−0.1–0.05)	0.416		Ischemia time*(*n* = *55*)	0.87	0.016/min	
Weight		98	0.06/kg (−0.091–0.231)	0.428		Weight*(*n* = *98*)	0.26	+0.09/kg	
Age		24	0.17/wk (−0.116–0.449)	0.234		Age*(*n* = *24*)	0.63	−0.18/wk	

NS = non significant. *Variable was added to the multivariable model separately, due to missing data. Total multivariable meta-regression was significant (p = 0.0001). n = the number of comparisons (=113 in total).

### Mortality

Univariable meta-regression showed no variables investigated correlated with peri-procedural mortality (Table 5). The subsequent multivariable meta-regression was non-significant (p = 0.33), so we did not proceed with further post-hoc testing. A sensitivity analysis, which omitted all animals that were excluded for VF with no attempt to treat the arrhythmia, was performed and also did not show any correlation with the variables of interest, both uni- and multivariably.

**Table 5:** Univariable and multivariable meta-regression for peri- and post-procedural mortality.

Univariable Analysis							Multivariable analysis			
Variable	categories	% mortality peri-proc (n)	p-value	% mortality post-proc (n)	p-value	post-hoc p-value	Variable	p-value peri-proc	p-value post-proc	beta
Species	Dog	17.8% (93)	0.26	5.4% (122)	0.95		Species	NA	NA	
	Pig	14.6% (68)		5.1% (41)						
	Sheep	20.3% (9)		4.5% (2)						
Sex	Male	15.2% (63)	0.24	5.3% (39)	0.87		Sex	NA	NA	
	Female	13.7% (24)		5.% (25)						
	Both	19.8% (41)		5.9% (59)						
	Unknown	18.0% (41)		4.1% (30)						
Immunosupp	yes	*0%* (1)	0.23	0% (2)	0.44		Immunosupp	NA	NA	
	no	16.*8%* (169)		5.3% (152)						
Co-medication	yes	10.9% (7)	0.27	5.7% (7)	0.90		Co-medication	NA	NA	
	no	16.9% (163)		5.2% (147)						
Open vs closed model	Open	16.1% (118)		5.1% (105)	0.78		Open vs closed model	NA	NA	
	Closed	18.0% (52)		5.6% (49)						
Occlusion	Permanent	17.0% (43)	0.24	6.2% (39)	**0.005**	perm vs temp = 0.361	Occlusion	NA	NA	
	Temporary	16.9% (125)		4.6% (114)		**perm vs unknown = 0.003**				
	Unknown	0% (2)		34.8% (1)		**temp vs unknown = 0.002**				
Occluded vessel	LAD	16.8% (116)	0.72	4.8% (102)	0.72		Occluded vessel	NA	NA	
	LCX	15.9% (51)		6.4% (48)						
	LAD/LCX	26.9% (2)		3.7% (3)						
	Unknown	22.2% (1)		0% (1)						
Study Quality		−0.77/point (170)	0.28	−0.024/point (154)	0.96		Study Quality	NA	NA	
Follow-up duration		−0.004/hr (166)	0.78	0.0023/hr (152)	**0.03**		Follow-up duration (n = 113)*	NA	<**0.001**	**0.007/hour**
Ischemia time		0.006/min (123)	0.10	−0.002/min (114)	0.52		Ischemia time (*n* = 1*13*)*	NA	0.77	−0.0007/min
Weight		−0.1/kg (153)	0.29	−0.06/kg (138)	0.30		Weight(*n* = *153*)*	NA	NA	
Age		+0.06/wk (15)	0.79	−0.015/wk (12)	0.92		Age(*n* = *15*)*	NA	NA	

*Variables added to the multivariable model separately, due to missing data. Multivariable meta-regression was not significant (p = 0.33 and p = 0.42). Multivariable meta-regression with the addition of ischemia time was significant for post-procedural mortality (p = 0.04). n = the number of comparisons (=170 and 165 in total).

Univariable meta-regression for post-procedural mortality showed a correlation with follow-up time, with the addition of 0.002% per hour extra follow-up (p = 0.03). Multivariably, meta-regression was not significant and no further post-hoc analyses were done (p = 0.41). The selected multivariable regression with the addition of ischemia duration (which only applies to temporary occlusion models) was significant (p = 0.047) and post-hoc testing revealed follow-up time as the only significant independent predictor of post-procedural mortality (0.007%/hour, p = 0.001) in studies using a temporary occlusion model.

### Post-hoc sensitivity analyses for different pig strains

In a post-hoc analysis we compared the strains ‘regular pigs’, ‘minipigs’ and ‘unknown strains’ within the species group of pigs. For IS/AAR, univariable metaregression was significant (p = 0.025), due to a difference between the unknown group and regular pigs (+13.2% if unknown, p = 0.018) and the unknown group and minipigs (+25.7 if unknown, p = 0.048). There was no univariable difference for pigs vs minipigs (p = 0.32). These significant differences disappeared in multivariable meta-regression (p = 0.15 for the strain variable) (n = 2, 24 and 15 respectively for studies using minipigs, pigs and unknown strains). For the outcomes IS/LV (n = 14, 24 and 14) and ejection fraction (n = 28, 40 and 19) there was no significant difference between minipigs, pigs and unknown strains in both univariable (p = 0.84 and p = 0.91 respectively) and multivariable (p = 0.17 and p = 0.49 respectively) analysis.

### Prediction of outcomes in common large animal MI models

Predicted outcomes for predefined commonly used models were generated (Table 6), showing clear differences for all outcomes between these models.

**Table 6:** Predicted regular outcomes for common large animal MI models.

	Infarct size/Area at Risk	Infarct size/Left Ventricle	Ejection Fraction
**Pig I/R (60 min) LAD model**			
*1 day*	60%	19%	—^†^
*1 week*	55%	18%	42%
4 *weeks*	(37%)*	18%	42%
**Dog I/R (60 min) LAD model**			
1* day*	40%	15%	—^†^
*1 week*	35%	15%	36%
4 *weeks*	(18%)*	14%	36%
**Pig permanent LAD model**			
1* day*	88%	24%	—^†^
*1 week*	82%	24%	38%
4 *weeks*	(60%)*	24%	38%

*Assuming linear effect of follow-up duration. ^†^Not calculated due to few measurements and myocardial stunning.

## Discussion

The current meta-analysis systematically reveals the effect of methodological choices on primary outcome measurements in large animal MI studies. The identification of the effect of the different experimental setups is of great importance, since it will guide adequate expectations of study results and mortality for specific models. It also enables more adequate and precise power calculations, which are essential when designing any preclinical study. We can now quantify biological differentiating variables for certain effect sizes and more accurately determine if these models resemble human disease. We confirmed some known biological variability within these models, showed effects that can be translated to the human situation and were able to quantify these variations in a meta-analytic manner.

The different disease manifestation across **species** has been demonstrated in the past^17^, with canine hearts forming more collaterals than hearts of other species, which we broke down to a ~20% smaller IS/AAR for dog models compared to pig models and lower EF in sheep compared to pigs. Despite the ~20% smaller IS/AAR in dogs, EF does not differ between dogs and pigs. Supplemental Figure 5 shows the absence of a correlation between IS/AAR and EF in our dataset, which could possibly be explained by confounding factors, including follow-up time and occluded vessel. Since infarct size decreases over time^18^ (Table 6), cardiac remodeling affects ejection fraction by progressive dilatation and systolic dysfunction. Moreover, occlusion of the LAD results in a loss of apical contractility, leading to a more severe decrease of ejection fraction compared to LCx occlusion^19,20^. As data on age and weight was scarse, we conducted an extra sensitivity analysis to compare minipigs and pigs, as these are considered the same species, but differ substantially in terms of age and weight. In this analysis, we could not find a difference between the two, arguing that MI models in both strains behave similar in terms of regular outcomes.

Conserved within evolution, females seem to show smaller infarcts compared to mixed groups and male counterparts, which is in line with the clinical data on **sex influence** on infarct size, favoring female subjects^21–23^. Of note, using female animals might leave researchers with a smaller therapeutic window in infarct size, potentially explaining the reduced efficacy of anti-inflammatory compounds in female animals^8^. Interestingly, pump function seems more decreased in female animals, once again arguing that the different sexes do not respond completely similar to cardiac damage and subsequent remodeling. In this perspective, it is crucial for translational success to include both sexes in future preclinical research, as is also called for by the NIH in preclinical projects^24^. Furthermore, there seem to be fewer studies using (only) female animals in our dataset, potentially explaining why not all comparisons to the female group always reached statistical significance.

The observed difference of ~9% in IS/AAR for **different occlusion sites** (LAD vs LCX) is in line with the observed greater loss of regional systolic function for anterior wall ischemia^25^, but was not observed for the outcome EF.

The observed reduction of infarct size and EF when increasing **follow-up time** is interesting both from a methodological and biological point of view. Smaller infarct sizes might imply smaller therapeutic windows for new interventions, while a larger reduction in EF might account for the inverse reasoning. Biologically this might be explained by infarct resorption and subsequent myocardial wall thinning, resulting in a decreased attribution of the thinned scar to the total myocardial mass^18^. Other explanations could be possible regeneration and post-infarction hypertrophy. Hibernating myocardium is not likely to explain this phenomenon, as function should increase after myocardial stunning and hibernation in the early stages of an infarct. Regardless of the cause, a longer follow-up could lead to more clinically relevant conclusions and might need more power to show any true differences. Incorporation of regular MI **co-medication** also seems to reduce the IS/AAR, which might be crucial for clinically relevant translation to the same poly-pharmaceutical human situation. A limitation of this variable is of course bundling of all studies using one or more of these compounds for power-reasons; we are not able to pinpoint these effects to one single compound. However, for many of these compounds there is either preclinical or even clinical evidence that they can influence infarct size and other outcomes after MI and therefore might be relevant to take into account for future experimental study design^26,27^.

The addition of **quantification method** seems crucial to be able to correct for the effects that these have on our different outcomes. For ejection fraction especially, it is known that echocardiography can overestimate cardiac function compared to for example MRI^28^. Our analyses confirm this, making it crucial to correct for these methods in multivariable analyses.

Interestingly, the composition of the dataset blurred the effect of multiple variables in the univariable analysis for IS/AAR, while our multivariable approach revealed certain effects that would otherwise have gone unnoticed.

No difference in outcome was observed for **open versus closed modeling of MI**, in contrast to what has been demonstrated in a recent study^29^. This might mean that conclusions from certain experiments can only be applied to the same setting; in this case an ischemia-reperfusion pig model. On the other hand, it might imply that meta-analyses cannot reveal all subtle differences within MI animal models. The same holds true for other variables in our dataset, like **immunosuppression**, which theoretically could have an effect on all our outcomes of interest.

Furthermore, we are limited by the data we were able to extract. In preclinical meta-analyses, many ‘known unknowns’ are present; variables that one would like to analyze, but are not reported as such. This is resembled by the unexplained heterogeneity (for multivariable IS/AAR analysis R^2^ = ~46% and I^2^ = ~96%) that, in the case of our MI analyses, is potentially influenced by for example the specific occlusion site of the vessel (which directly influences the area at risk), weight of the animal or experience of the surgeon. However, with the variables available, we were able to explain a significant part of the observed heterogeneity, with model-specific differences and human-like variability for sex and co-medication.

Modeling mortality in our study did not result in many explanatory variables, so we can only give summary estimates based on the meta-analysis of the total data. On average, peri-procedural mortality was ~17%, while post-procedural mortality was condensed in a ~5% mortality rate. These are important numbers for future study designs, as power analyses are crucial in the success chance of (pre)clinical trials and the reduction of both type I and type II errors. It is possible that these numbers are incomplete or biased in the current analysis, due to incomplete reporting in prior studies. This might be less of a problem for future similar analyses as the reporting of animal studies will hopefully improve substantially due to the ARRIVE guidelines, EDA application and journals demanding complete reporting^30,31^.

The need for meta-research on methods and reproducibility has been solicited for by the community and is a crucial process in the self-cleansing ability of research^10^. This paper untangled a part of the variation observed and generates realistic starting points for well-needed large animal MI models, hopefully adding further insight in disease understanding, accurate modeling of MI and more translational success for new cardiac interventions.

Being able to explain and predict a ‘point of departure’ in large animal MI models will prove useful to tailor experiments and make reasonable power calculations based on the expected damage, mortality and potential experimental effect (example in Fig. 1). This will potentially result in more accurately powered studies, more definite answers to research questions and less waste of animal lives and research money^32^. Many clinically relevant patient characteristics seem to be of influence in the preclinical setting, and will potentially influence any outcome if not taken into account. In the current era of translational science, all researchers need to take this variation into account when designing new studies to optimize the chance of success of any large animal experiment.

## Electronic supplementary material

Supplementary Material
