# Publisher Correction: Optimization of large animal MI models; a systematic analysis of control groups from preclinical studies

**DOI:** 10.1038/s41598-018-23615-9

**Published:** 2018-04-11

**Authors:** P. P. Zwetsloot, L. H. J. A. Kouwenberg, E. S. Sena, J. E. Eding, H. M. den Ruijter, J. P. G. Sluijter, G. Pasterkamp, P. A. Doevendans, I. E. Hoefer, S. A. J. Chamuleau, G. P. J. van Hout, S. J. Jansen of Lorkeers

**Affiliations:** 1 Department of Cardiology, University Medical Center Utrecht, Utrecht, The Netherlands; 2 Center for Clinical Brain Sciences, University of Edinburgh, Edinburgh, United Kingdom; 3 Hubrecht Institute, Koninklijke Nederlandse Academie van Wetenschappen (KNAW), University Medical Center Utrecht, Utrecht, The Netherlands; 4 Netherlands Heart Institute (ICIN), Utrecht, The Netherlands; 5 UMC Utrecht Regenerative Medicine Center, University Medical Center Utrecht, Utrecht, The Netherlands; 6 Central Military Hospital, Utrecht, The Netherlands; 7 Department of Clinical Chemistry and Hematology, University Medical Center Utrecht, Utrecht, The Netherlands

Correction to: *Scientific Reports* 10.1038/s41598-017-14294-z, published online 27 October 2017

In the original version of this Article, the author S. J. Jansen of Lorkeers was incorrectly indexed. This error has now been corrected.

