# Supplementary material for: Optimization of large animal MI models; a systematic analysis of control groups from preclinical studies
Source: Sci Rep. 2017 Oct 27;7:14218. doi: 10.1038/s41598-017-14294-z (PMC5660150; doi:10.1038/s41598-017-14294-z)
Supplement: Supplementary file 1 — Supplementary Material [file 41598_2017_14294_MOESM1_ESM.pdf]

Optimization of large animal MI models; a systematic analysis of control groups from preclinical studies

P.P. Zwetsloot, L.H.J.A. Kouwenberg, E.S. Sena, J.E. Eding, H.M. den Ruijter, J.P.G. Sluijter, G. Pasterkamp, P.A. Doevendans, I.E. Hoefer, S.A.J. Chamuleau, G.P.J. van Hout* & S.J. Jansen of Lorkeers*

* contributed equally

Supplemental Methods

<u>Extraction of mortality data</u>

Results on peri- and post-procedural mortality were extracted for all studies; peri-procedural meaning within the timeframe of the infarct-induction process ('death during surgical procedures') and post-procedural meaning after the disease-inducing procedure. Mortality, if mentioned, was extracted for control animals, unless peri-procedural mortality was assessed before group assignment; in that case all study animals were included since potential benefit or harm of any intervention was excluded. Hence, the number of analyzed animals for peri-procedural mortality is will be larger than the number of analyzed animals for certain standard outcome measures. In the case of mentioning mortality both pre- and post-randomization within the setting of peri-procedural mortality, both were recorded and converted to one single value. If peri-procedural mortality was not specifically mentioned, we excluded the paper from our analyses. If the number of animals after the procedure was the same as in the final assessment, we assumed no mortality. Furthermore, if measurements for the primary outcome were done in fewer animals than at the start of the experiment without explaining the loss of these animals, we excluded these comparisons for our mortality analyses. Any procedural complications not due to the induction of the myocardial infarction (MI) itself were not counted as 'natural' mortality. Due to evolving methodology over time in MI modeling with regards to the treatment of ventricular fibrillation (VF) during induction of MI, we recorded if animals were treated for VF (either by medication or defibrillation) or were excluded immediately and performed a predefined sensitivity analysis to exclude a potential effect of this specific early exclusion.

Supplementary Figure 1

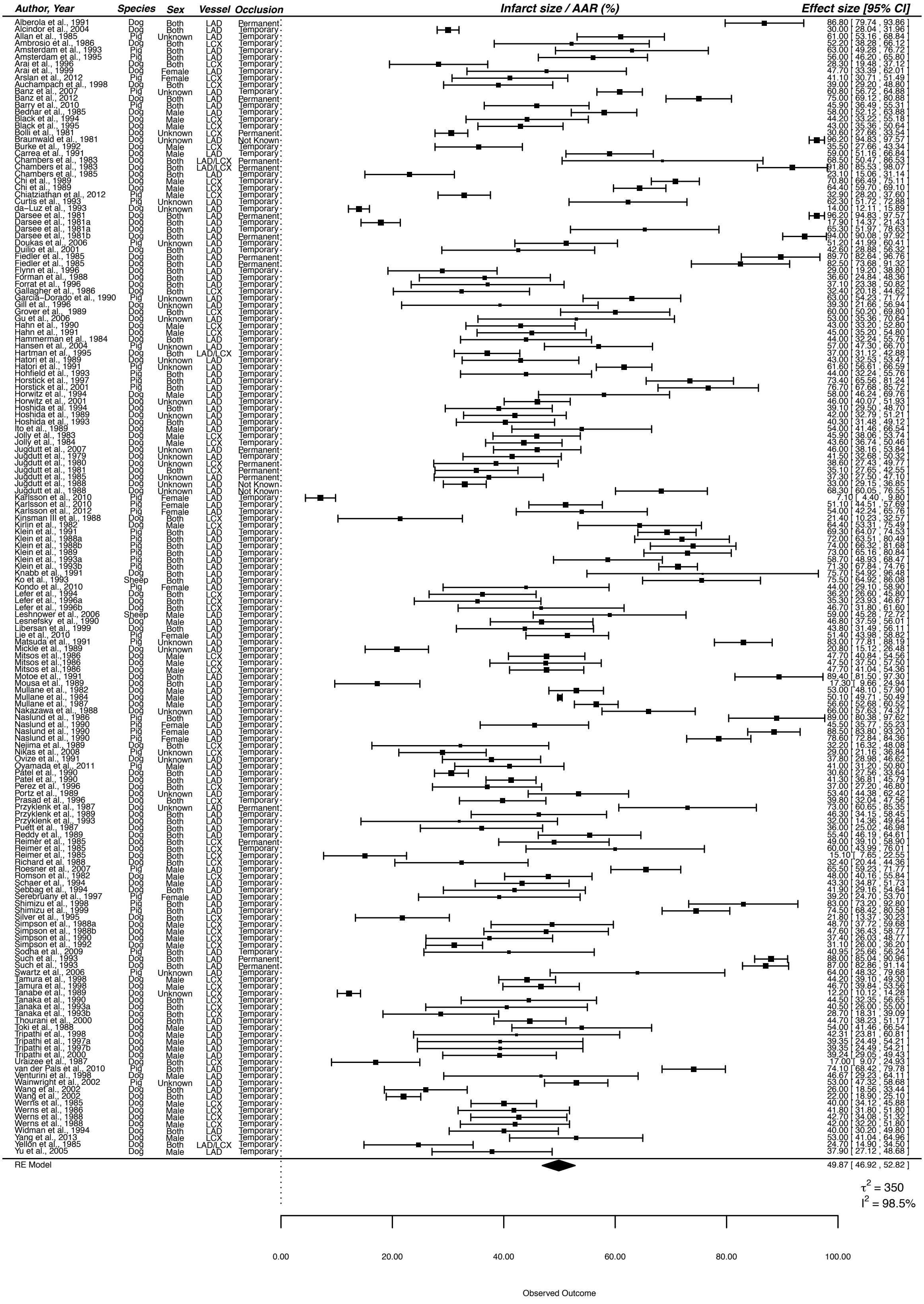

Supplementary Figure 2

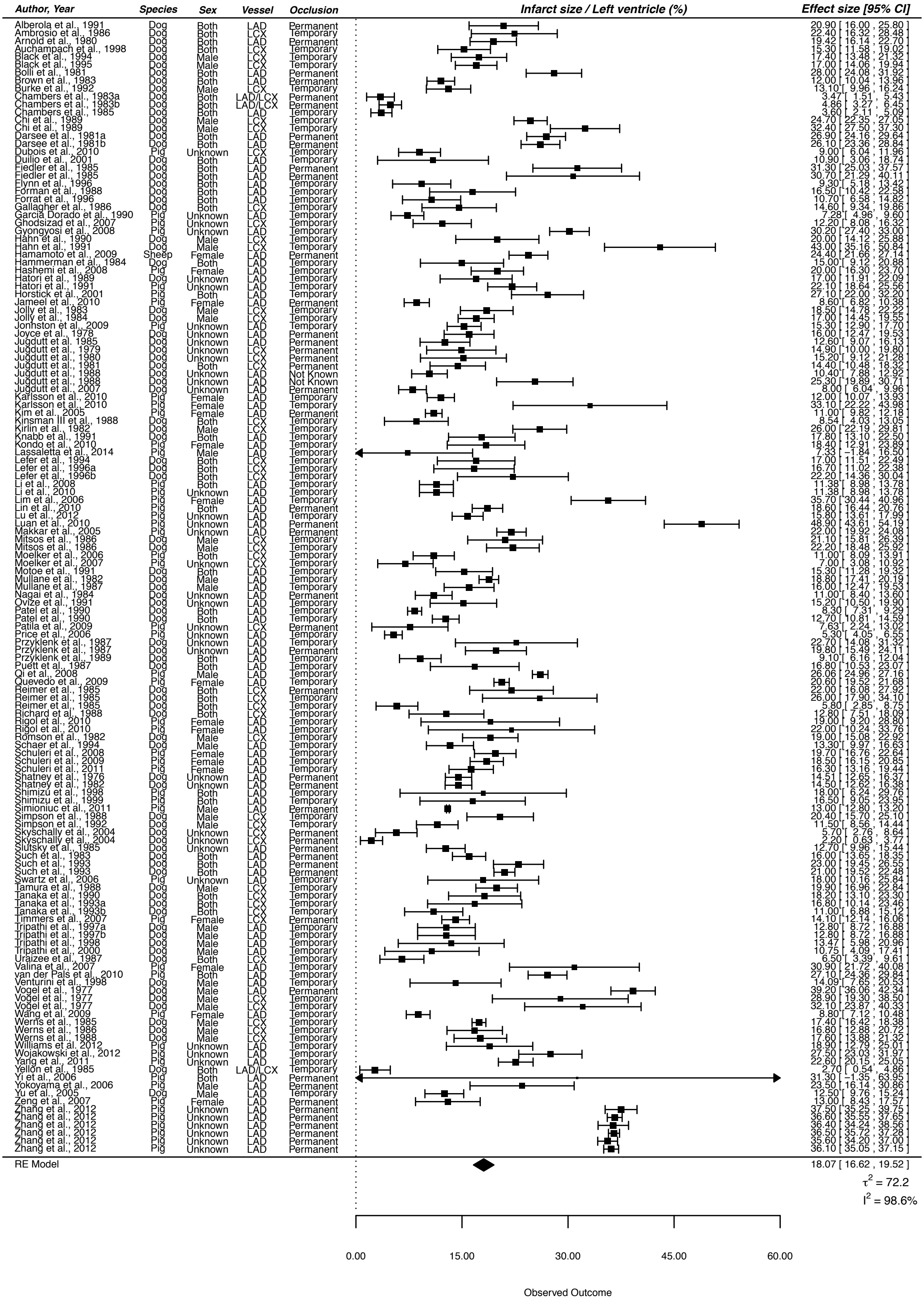

Supplementary Figure 3

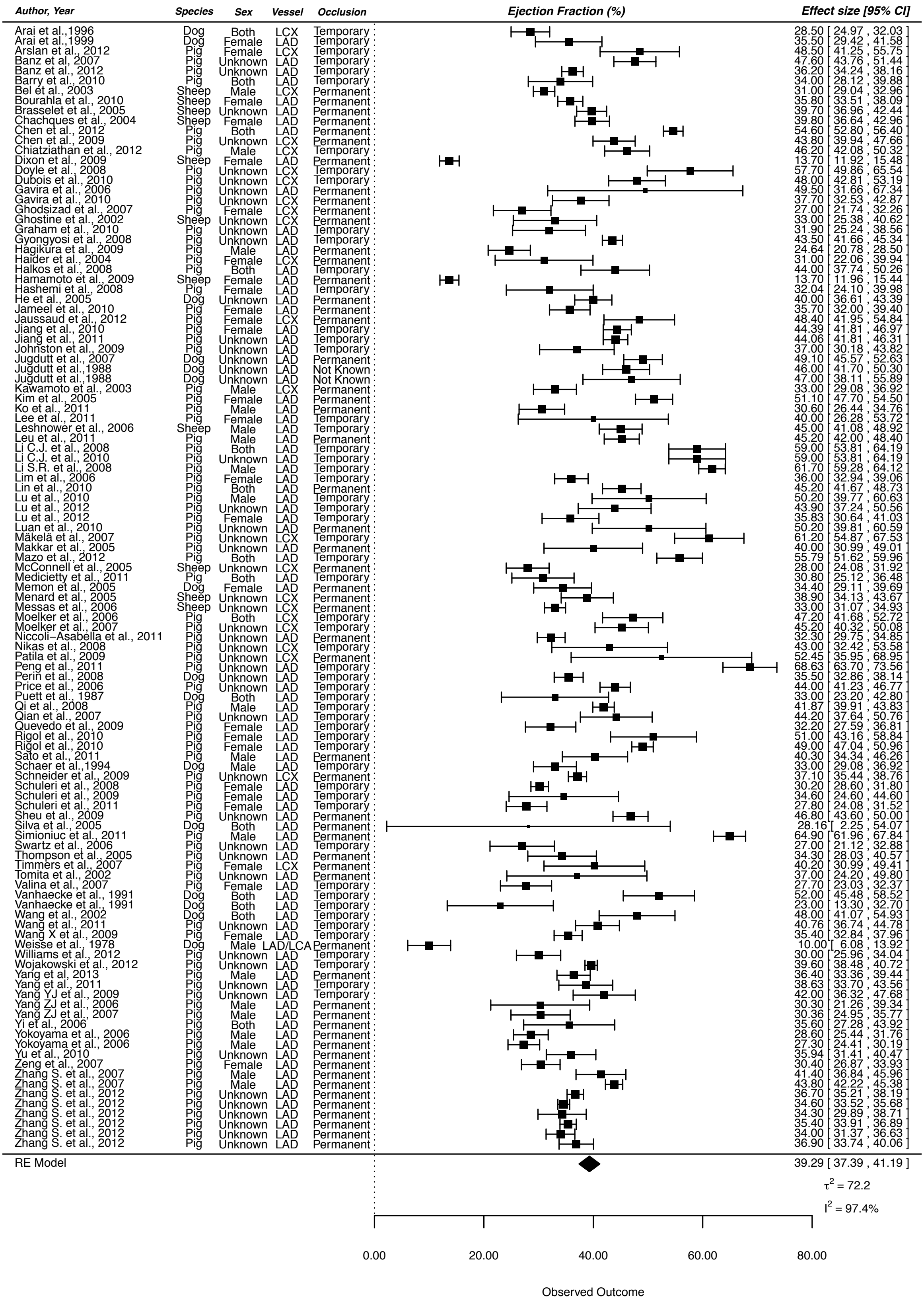

Supplementary Figure 4

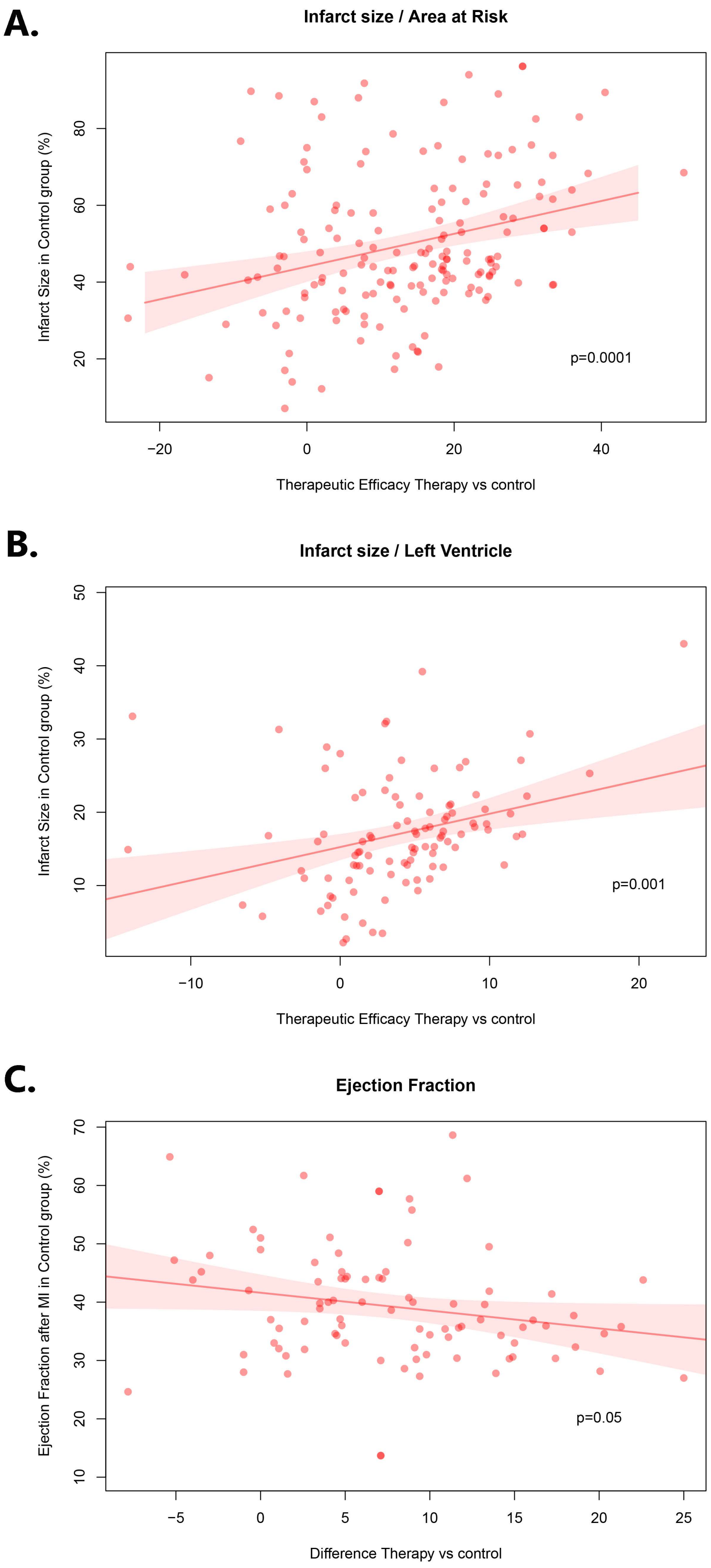

Supplementary Figure 5

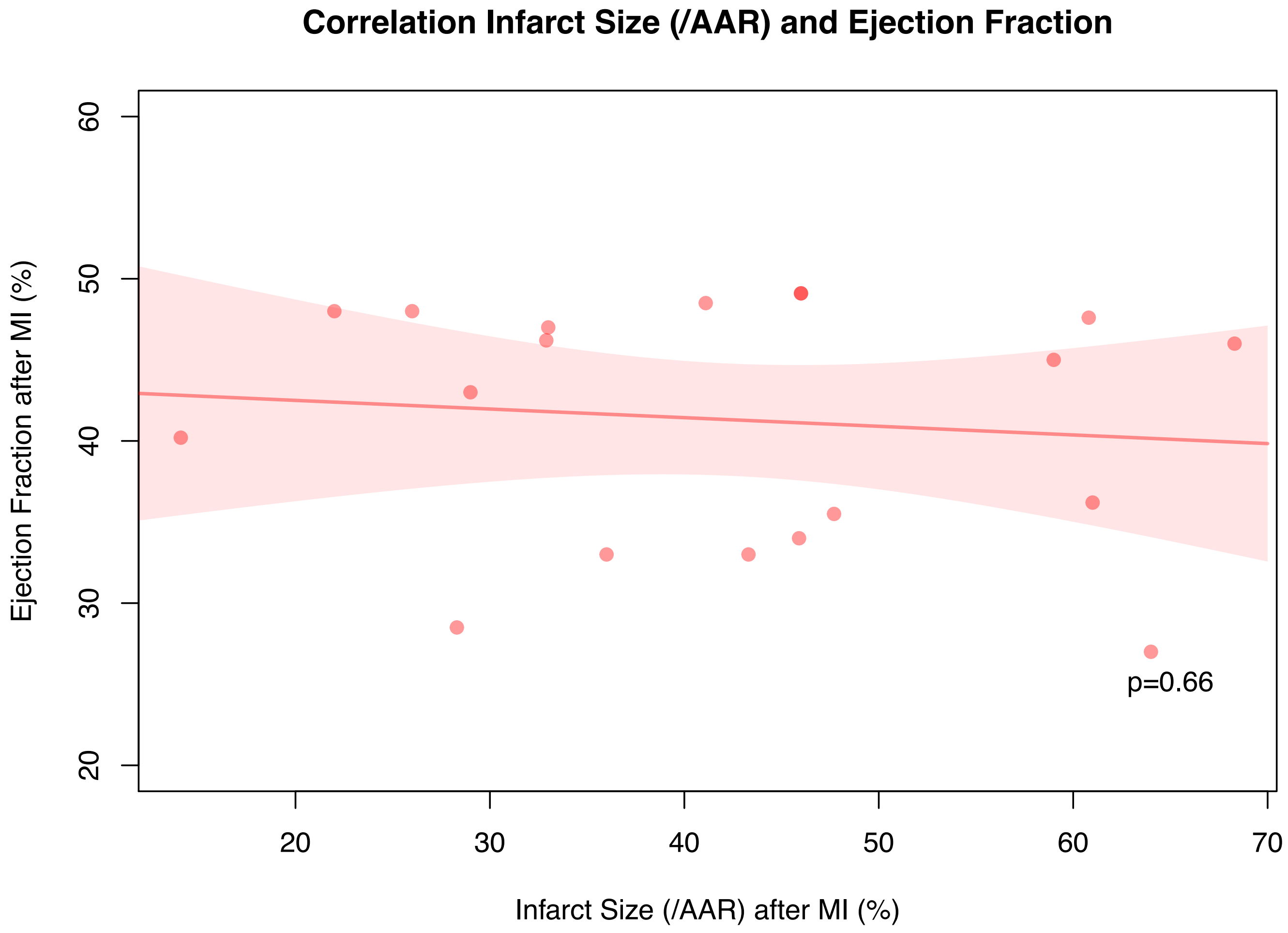

Supplemental Figure Legends

Supplemental Figure 1: Forest plot of all control groups from included studies measuring IS/AAR.

Supplemental Figure 2: Forest plot of all control groups from included studies measuring IS/LV.

Supplemental Figure 3: Forest plot of all control groups from included studies measuring EF.

Supplemental Figure 4: Regression analyses for the reported outcome in the control groups and the actual effect size (difference between therapy and control) in different studies for (A) IS/AAR, (B) IS/LV and (C) EF.

Supplemental Figure 5: Regression analysis for IS/AAR and EF, when both are measured in included studies.

R code used for figures and part of the analyses

```
library(xlsx)
library(metafor)

setwd("/")

read.xlsx("working_Antiinflammatory compounds in large
 animals_Cleaned_121114_take2.xls",sheetIndex=4)->ISAAR

read.xlsx("working_Antiinflammatory compounds in large
 animals_Cleaned_121114_take2.xls",sheetIndex=8)->ISLV

read.xlsx("working_Stem Cells in large animal MI_Sanne.xls",sheetIndex=2)->EF

summary(lm(Mean.in.Control.Group~rawmeandifferencex1x2, data=ISAAR))

plot(Mean.in.Control.Group~rawmeandifferencex1x2, data=ISAAR)




pdf("ISAAR controls vs efficacy.pdf",height=6,width=8)

plot(Mean.in.Control.Group~rawmeandifferencex1x2,
 data=ISAAR,col=rgb(1,0,0,0.4),pch=19,xlab="Therapeutic Efficacy Therapy vs
 control",ylab="Infarct Size in Control group (%)")
lm(Mean.in.Control.Group~rawmeandifferencex1x2, data=ISAAR)->mod1
predict(mod1,newdata=data.frame(rawmeandifferencex1x2=-22:45),se=T)->mod1preds
lines(-22:45, mod1preds$fit,col=rgb(1,0,0,0.4),lwd=2)
polygon(c(-22:45,45:-22),c(mod1preds$fit+1.96*mod1preds$se,rev(mod1preds$fit-
 1.96*mod1preds$se)),lty=0,col=rgb(1,0,0,0.1))
text(40,20,c("p=0.0001"))
title("Infarct size / Area at Risk")


dev.off()

summary(lm(Mean.in.Control.Group~rawmeandifferencex1x2, data=ISLV))

plot(Mean.in.Control.Group~rawmeandifferencex1x2, data=ISLV)

pdf("ISLV controls vs efficacy.pdf",height=6,width=8)

plot(Mean.in.Control.Group~rawmeandifferencex1x2,
 data=ISLV,col=rgb(1,0,0,0.4),pch=19,xlab="Therapeutic Efficacy Therapy vs
 control",ylab="Infarct Size in Control group (%)")
lm(Mean.in.Control.Group~(rawmeandifferencex1x2), data=ISLV)->mod1
predict(mod1,newdata=data.frame(rawmeandifferencex1x2=-22:45),se=T)->mod1preds
lines(-22:45, mod1preds$fit,col=rgb(1,0,0,0.4),lwd=2)
polygon(c(-22:45,45:-22),c(mod1preds$fit+1.96*mod1preds$se,rev(mod1preds$fit-
 1.96*mod1preds$se)),lty=0,col=rgb(1,0,0,0.1))
text(20,10,c("p=0.001"))
title("Infarct size / Left Ventricle")


dev.off()
```

```
summary(lm(Mean.in.Control.Group~Effect.Size, data=EF))

plot(Mean.in.Control.Group~Effect.Size, data=EF)

pdf("EF controls vs efficacy.pdf",height=6,width=8)

plot(Mean.in.Control.Group~Effect.Size,
 data=EF,col=rgb(1,0,0,0.4),pch=19,xlab="Difference Therapy vs
 control",ylab="Ejection Fraction after MI in Control group (%)")
lm(Mean.in.Control.Group~(Effect.Size), data=EF)->mod1
predict(mod1,newdata=data.frame(Effect.Size=-22:45),se=T)->mod1preds
lines(-22:45, mod1preds$fit,col=rgb(1,0,0,0.4),lwd=2)
polygon(c(-22:45,45:-22),c(mod1preds$fit+1.96*mod1preds$se,rev(mod1preds$fit-
 1.96*mod1preds$se)),lty=0,col=rgb(1,0,0,0.1))
text(20,20,c("p=0.05"))
title("Ejection Fraction")


dev.off()


#### CORRELATIONS EF vs ISAAR

read.xlsx("working_Antiinflammatory compounds in large
 animals_Cleaned_121114_take2.xls",sheetIndex=1)->correlations

summary(lm(IS~Mean.EF, data=correlations))

pdf("correlation IS vs EF.pdf",height=6,width=8)

plot(Mean.EF~IS, data=correlations,col=rgb(1,0,0,0.4),pch=19,xlab="Infarct Size
 (/AAR) after MI (%)",ylim=c(20,60),ylab="Ejection Fraction after MI (%)")
lm(Mean.EF~(IS), data=correlations)->mod1
predict(mod1,newdata=data.frame(IS=-10:70),se=T)->mod1preds
lines(-10:70, mod1preds$fit,col=rgb(1,0,0,0.4),lwd=2)
polygon(c(-10:70,70:-10),c(mod1preds$fit+1.96*mod1preds$se,rev(mod1preds$fit-
 1.96*mod1preds$se)),lty=0,col=rgb(1,0,0,0.1))
text(65,25,c("p=0.66"))
title("Correlation Infarct Size (/AAR) and Ejection Fraction")

dev.off()


### FOREST PLOTS ###
###ISAAR###

ISAARforest<-rma(yi= Mean.in.Control.Group,sei=SE,data=ISAAR)
str(ISAARforest)
## ALL STUDIES FOREST PLOT ##
pdf("ISAARforest.pdf",width=8,height=12)

forest (ISAARforest,xlim=c(-50, 115), slab=as.character(ISAAR$AuthorYear),
 ilab=cbind(as.character(ISAAR$species),as.character(ISAAR$sex),
```

```
 as.character(ISAAR$vessel_occluded),as.character(ISAAR$occlusion_IR)),ilab.xpo
 s=c(-26,-19,-12,-4))
text(-50,167,"Author, Year",font=4,cex=0.5,pos=4)

text(-26,167,"Species",font=4,cex=0.5)
text(-19,167,"Sex",font=4,cex=0.5)
text(-12,167,"Vessel",font=4,cex=0.5)
text(-4,167,"Occlusion",font=4,cex=0.5)
text(50,167,"Infarct size / AAR (%)",font=4,cex=0.6)
text(115,167,"Effect size [95% CI]",font=4,cex=0.6,pos=2)
text(102,-4.2,expression(tau^2~"= 350"),cex=0.6,pos=4)
text(102,-6.5,expression("I"^2~"= 98.5%"),cex=0.6,pos=4)

dev.off()

## ISLV##
ISLVforest<-rma(yi= Mean.in.Control.Group,sei=SE,data=ISLV)
str(ISLVforest)
## ALL STUDIES FOREST PLOT ##
pdf("ISLVforest.pdf",width=8,height=12)

forest (ISLVforest,xlim=c(-50, 80), slab=as.character(ISLV$AuthorYear),
 ilab=cbind(as.character(ISLV$species),as.character(ISLV$sex),
 as.character(ISLV$vessel_occluded),as.character(ISLV$occlusion_IR)),ilab.xpos=
 c(-28,-21,-14,-6), alim=c(0,60))
text(-50,145,"Author, Year",font=4,cex=0.5,pos=4)
text(-28,145,"Species",font=4,cex=0.5)
text(-21,145,"Sex",font=4,cex=0.5)
text(-14,145,"Vessel",font=4,cex=0.5)
text(-6,145,"Occlusion",font=4,cex=0.5)

text(35,145,"Infarct size / Left ventricle (%)",font=4,cex=0.6)
text(80,145,"Effect size [95% CI]",font=4,cex=0.6,pos=2)
text(70,-3.2,expression(tau^2~"= 72.2"),cex=0.6,pos=4)
text(70,-5.5,expression("I"^2~"= 98.6%"),cex=0.6,pos=4)

dev.off()


## EF ##
EFforest<-rma(yi= Mean.in.Control.Group,sei=SE,data=EF)
str(EFforest)

pdf("EFforest.pdf",width=8,height=12)

forest (EFforest,xlim=c(-68, 100), slab=as.character(EF$AuthorYear),
 ilab=cbind(as.character(EF$Animal),as.character(EF$Sex),as.character(EF$Locati
 on.of.Infarct),as.character(EF$Type.of.Ischaemia)),ilab.xpos=c(-33,-24,-16,-
 6))
text(-68,115,"Author, Year",font=4,cex=0.5,pos=4)
text(-33,115,"Species",font=4,cex=0.5)
text(-24,115,"Sex",font=4,cex=0.5)
text(-16,115,"Vessel",font=4,cex=0.5)
text(-6,115,"Occlusion",font=4,cex=0.5)
```

```
text(35,115,"Ejection Fraction (%)",font=4,cex=0.6)
text(100,115,"Effect size [95% CI]",font=4,cex=0.6,pos=2)
text(80,-3.2,expression(tau^2~"= 72.2"),cex=0.6,pos=4)
text(80,-5.5,expression("I"^2~"= 97.4%"),cex=0.6,pos=4)

dev.off()

###

help(forest)
```
